# Who sifts? Knowledge as a public good in the age of AI

**DOI:** 10.1371/journal.pgph.0007381

**Published:** 2026-10-21

**Authors:** Kent Buse, Sarah Hawkes

**Affiliations:** 1 Global 50/50, Cambridge, United Kingdom; 2 Monash University Malaysia, Kuala Lumpur, Malaysia; PLOS: Public Library of Science, UNITED STATES OF AMERICA

## Abstract

The institutions that turned information into evidence for public policy decisions are being defunded and discredited just as generative AI floods them with fluent, unsourced text. Sifting what is known from what is claimed is now a core responsibility for public health institutions.

In February 2025 the United States ended its funding of the Demographic and Health Surveys programme, which for four decades had produced the population trends, child mortality, nutrition, immunisation and other health estimates on which ministries, UN agencies, and researchers in more than 90 countries relied. Surveys were underway or ready in 23 countries when the money stopped. The annual cost had been about US$47 million, less than 0.1% of the USAID budget [1]. Private philanthropy has since funded a reduced programme. The backbone of population health data for much of the world now rests on a time-limited grant.

In the same period, generative artificial intelligence (AI) entered the organisations whose job is to sift such data: to separate what has been measured from what is merely claimed. By late 2024 the UN system had catalogued more than 700 AI projects across some 50 entities, almost half of them text and language applications [2]. WHO launched its own generative health chatbot in 2024; an early assessment found operational failures that risked its credibility rather than strengthening it [3]. Ask any large language model for a stunting estimate for any district and it will supply one, fluently, instantly, and without provenance. We checked the status of the Demographic and Health Surveys programme while writing this piece; the first answer returned was an AI-generated summary, confident and only partly sourced. The machinery that produced verified knowledge is being dismantled just as machinery that produces plausible knowledge at negligible financial cost is being installed by the institutions whose job it was to tell the difference.

## Information management is about power

This sounds like information technology, but it is really a question of power. How WHO, UNICEF, a national public health institute, or a statistics office manages information determines whose evidence is admitted, what never reaches the policy agenda, and what publics and decision-makers come to regard as common sense. These are the three faces of power described by Bachrach and Baratz and by Lukes: visible decisions, control of the agenda, and the shaping of preferences [4,5]. The third face concerns us most, because its success is invisible. Nobody notices the question never asked.

The point is not new. Tobacco companies used their power to manufacture doubt for over half a century because doubt, unlike a counter-argument, needs only to persist [6]. The sugar industry funded research in the 1960s that turned scientific attention from sugar to fat, shaping dietary guidance for a generation [7]. Corporate lobbying of US positions towards WHO averaged US$12.5 million a year between 2006 and 2015, and part of its apparent purpose was to erode confidence in the institution [8]. Deploying the third face of power, redirecting what counts as evidence and knowledge, and discrediting those who certify it, is an old strategy. What is new is the cost, speed, and reach of the machinery.

A working distinction helps (see Fig 1). Information is whatever moves through the environment around us: data, text, testimony, rumour, and fabrication alike. Evidence is the subset of information that bears on the truth of a claim and can justify action. Knowledge is belief that is both true and justified. A fact is what makes a true claim true, whether or not anyone believes it. Public institutions exist, in part, to move material along that chain, sifting information into evidence and evidence into knowledge that can be acted on. The sifting is slow, contestable, and expensive, and rests on epistemic authority: the socially recognised standing to say what counts.

**Fig 1:**
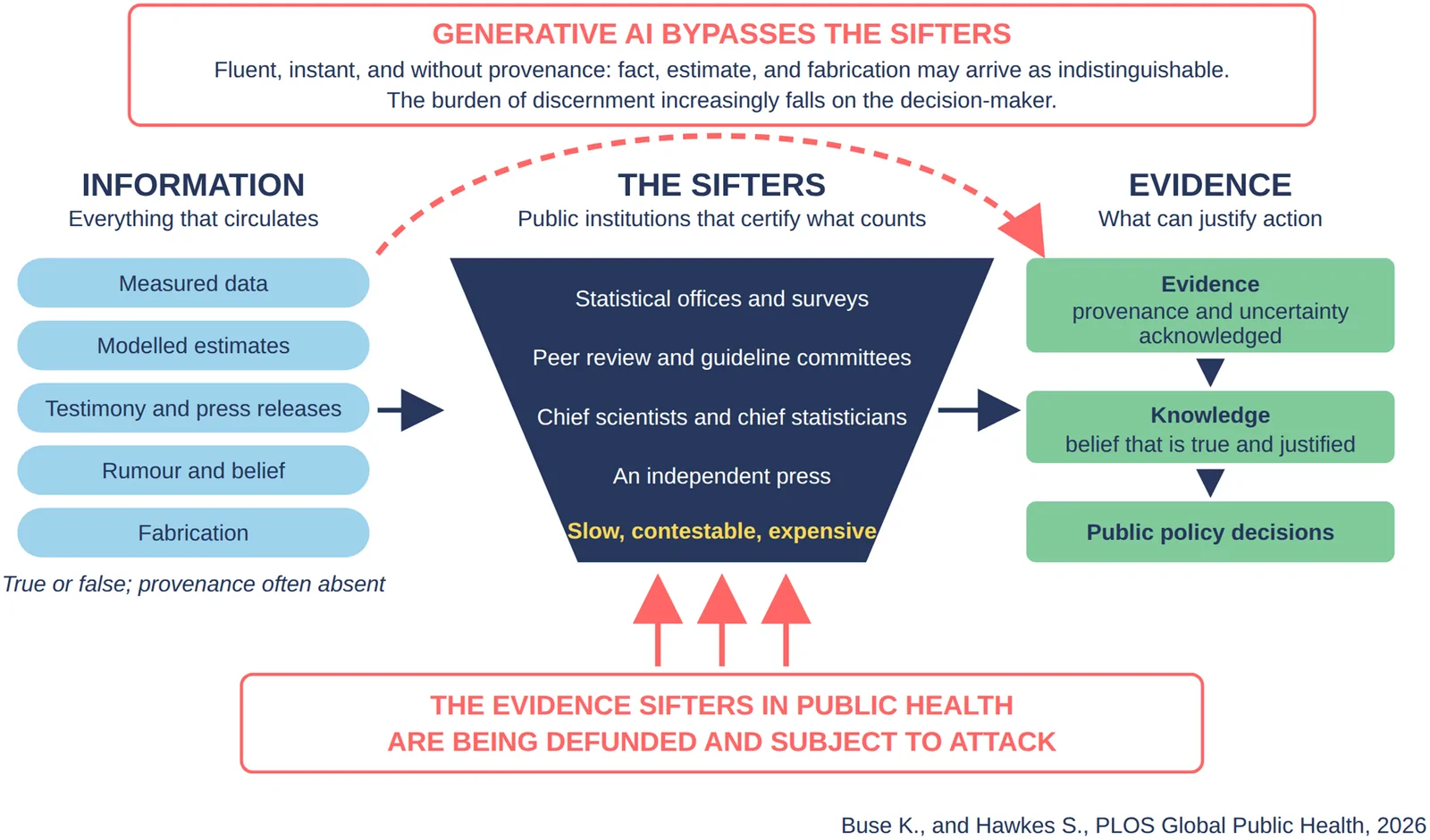
The abundance paradox: evidence in the era of AI.

Large language models act on every link of that chain. They generate abundance without discernment. In 1971 Herbert Simon, Nobel Laureate and pioneer of the field of attention economics, observed that a wealth of information creates a poverty of attention: the more there is to absorb, the less of it anyone can assess [9]. WHO named the pandemic version of the problem an infodemic [10].AI models now industrialise the problem. They strip provenance, presenting the peer-reviewed estimate, the modelled projection, the figure quoted in a press release, and the hallucination in the same fluent register. They can fabricate citations at rates that would end a research career [11]. Their training corpora reproduce the prevailing distribution of published knowledge with all its inherent biases: English language, Global North, and beset with conflicts of interest that shape findings [12].

That distinction between information and evidence was already under strain before the models arrived. Around half of the health-related Sustainable Development Goal indicators are now tracked primarily through modelled estimates rather than data reported by countries, in part because many lack the capacity and resources for ongoing health surveillance [13]. Modelled estimates are information. Identifying the evidence within estimates has traditionally been the responsibility of scientists not policy-makers. But at a time when the authority of scientists, statisticians, and an independent press is under deliberate attack by those who benefit from an evidence-free process, policy-makers are left to resolve the information/evidence distinction for themselves [14].

## Capacitating the sifters

The sifting cannot be left to the platforms or the market. The firms that build the models sell fluency, not verification. Nor can it be left to individual users; discernment at the scale of a health system remains an institutional function. It belongs to public health agencies, research institutes including universities, statistical offices, and multilateral organisations, which alone hold a public mandate and, for now, the legitimacy to exercise it.

These institutions are not without sifters. WHO has a chief scientist, a guidelines review committee, and a science council; UNICEF has a chief statistician and a research office; the UN system has a chief statistician. But these functions were built to certify what an institution publishes, not to govern how thousands of staff now generate text and estimates with commercial tools, and they are being cut. WHO’s 2025 restructuring reduced director posts from 76 to around 34, with up to 30–40% of headquarters staff facing redundancy [15]. The AI guidance that WHO and the UN system have issued addresses the ethics and governance of the tools. We hope it will now add responsibility for information-sifting to these existing functions, along with the budgets to exercise it.

In this context, capacity has two dimensions. The first is technical. Agencies need provenance standards for anything they publish or generate with AI, showing whether a figure was measured, modelled, or generated, and with what uncertainty. They need living evidence functions that work at the speed of politics without abandoning verification, and renewed investment in primary data collection and national statistical capacity. A survey programme costing 0.1% of one donor’s aid budget should never again depend on that donor, or on a single foundation.

The second, more challenging dimension, is political. In an era of information-overload and attention-deficit we need explicit defence of the scientists and statisticians on whose work the public health institutions depend. Silence when they are attacked is itself an information management decision. Work that is independent, transparent and held to account must be the basis for providing evidence that is trusted by publics and policy-makers alike.

The post-truth condition is not a crisis that will pass. It is the terrain on which public institutions now operate and on which they will be judged. Those that learn to curate, contest, and defend knowledge as a public good will be the institutions still trusted a decade from now. The rest will have outsourced the question of what is true to whoever owns the model.
